# Supplementary material for: Rapid turnover of life-cycle-related genes in the brown algae
Source: Genome Biol. 2019 Feb 14;20:35. doi: 10.1186/s13059-019-1630-6 (PMC6374913; doi:10.1186/s13059-019-1630-6)
Supplement: Supplementary file 3 — Protein alignments, corresponding Gblocks html file and CDS sequences for the lineage conserved gametophyte-biased genes. (ZIP 2109 kb) [file 13059_2019_1630_MOESM3_ESM.zip › Con_GA_alignments/Ec-14_004320.aa_ali.fasta-gb.htm]

Ec-14\_004320.aa.fasta.trans.aa\_ali.fasta


## Gblocks 0.91b Results

Processed file: **Ec-14\_004320.aa.fasta.trans.aa\_ali.fasta**  
Number of sequences: **2**  
Alignment assumed to be: **Protein**  
New number of positions: **613** (selected positions are underlined in blue)

```
                         10        20        30        40        50        60
                 =========+=========+=========+=========+=========+=========+
Ec-14_004320     MATVELSVFGLGSQIDVTNETLALAGVVGFTVAFEMAAHRLEHRLAGTPYMGMLAKIYKE
MSTRG.49732.1    MATVPLSVFGLGSQVDITSETLALAGLVGFTVLFEMAAHRLEHRLEGTPYMGMLAKIYKE
                 ############################################################


                         70        80        90       100       110       120
                 =========+=========+=========+=========+=========+=========+
Ec-14_004320     LMIMGFISLGVFLLLQFGSVGSTLGFYTFEFAHIVIFFTTLIFVAQACLMMVLSSRLKRR
MSTRG.49732.1    LMIMGFISLGVFLTLQFGSVGSSVGFYTFEFAHIVIFCTTLMFVMQACLMMVLSSQLKRR
                 ############################################################


                        130       140       150       160       170       180
                 =========+=========+=========+=========+=========+=========+
Ec-14_004320     YEKSTNISVDHLLTFYSLHRKSKMFHARWLPVSDLREVMEFKIVHSFFRTTFALPTTFNF
MSTRG.49732.1    YEKSTNISVDHLLTFYSLHRKSKVFHARWLPTSDLREVMEFKIVYFFFRTTFALPTTFNF
                 ############################################################


                        190       200       210       220       230       240
                 =========+=========+=========+=========+=========+=========+
Ec-14_004320     ALYMRKATDSHILALVDVESSTWVVLGVVFFLNLARIKIWQMIGLTKEEEDH-ADDDHGT
MSTRG.49732.1    ALYMRQATDSHILALVDVESSTWIILAALIFLNLARIKIWQQIGATEEAEDYPTTDDHSP
                 ###################################################    #####


                        250       260       270       280       290       300
                 =========+=========+=========+=========+=========+=========+
Ec-14_004320     TDDHGSSSTSSHDDHVATTSSGYDNGRRWLAGDMGEFASDAYGNTGSDGETMFIVAGWLM
MSTRG.49732.1    TDDHTSS---ARD---------DDHRRRSLAGAGGDDGSGSYSNTGSDGELMFIVAGWLM
                 #######                #####################################


                        310       320       330       340       350       360
                 =========+=========+=========+=========+=========+=========+
Ec-14_004320     LALAVFNYFMSRWVELRLLKKAGCEDTHEYHNQLAVIEKQTLEAKKHENRRRTVLNRGGH
MSTRG.49732.1    LAMAAANYFMSRWVELRLLKKAGCEGTREYHIRLAEIEKQTLEAKREENERRTVVNGGGH
                 ############################################################


                        370       380       390       400       410       420
                 =========+=========+=========+=========+=========+=========+
Ec-14_004320     LQRLKRQVDEAKEDSRAHHS-------HHRHWRRKEGGGMDEKSFHASISRHLGQAMRNA
MSTRG.49732.1    LERLKRQVDKAKENGKGHHRRHHHRHHHHQHRLKKGDGHEDETDLHRSISRHLGQAKHHA
                 ###################        #################################


                        430       440       450       460       470       480
                 =========+=========+=========+=========+=========+=========+
Ec-14_004320     QALSSSVGSADIKEEIEKVIQRSTP-PRGFLRLGSGNRSKILPEEELNRNSTNERDLLVD
MSTRG.49732.1    QALYTRVGSGDLHKIAKNAIVRSSSSPSGTLFSRGGNGSKVVPG--------------VS
                 #######################   #################                 


                        490       500       510       520       530       540
                 =========+=========+=========+=========+=========+=========+
Ec-14_004320     GLGTTANRSVAELASKRGDTTPLPPSVPNDVGSSGSNTTATAAAAAAGASVALLPRAQPP
MSTRG.49732.1    GGSAQSSPGYQGLRAAAG--EAIGTTTTPKLGRNGDTETSHSSTLTHAGGVTRDALTTAT
                                                                             


                        550       560       570       580       590       600
                 =========+=========+=========+=========+=========+=========+
Ec-14_004320     GERADLGLSRGTAAAAAETGGGAREYTTVLVAPDPGDSVRASEYGVDSESRDDSSGNNRR
MSTRG.49732.1    YESGGGMSAGGQTGYLTQSGPGSR-------APDEVRTFRLSDYGLERELNSDASLADDG
                  #######################       ########################     


                        610       620       630       640       650       660
                 =========+=========+=========+=========+=========+=========+
Ec-14_004320     SPQEREGLD-VEEEVKEGDDVNRPAEEWGRG----GSWSGGGGRQVLGDDCHQLRYVMNI
MSTRG.49732.1    CRHRRYLKERINDTLIEVEDERRLESDGGGDQDSLGGFRDGRVAAASSENTQEDQFAVSH
                                 #############                               


                        670       680       690       700       710       720
                 =========+=========+=========+=========+=========+=========+
Ec-14_004320     PSLRTQ-------KDGGSSKDFTRSPLEVLVKREPKPLLQDKTVSHR--------AGYVC
MSTRG.49732.1    PSLLAPPGNGAGDKGSGEASELS-SPMQLVVKPGPTPLPEEKTATVHDRCRERLGSGGRG
                                         ###################                 


                        730       740       750       760       770       780
                 =========+=========+=========+=========+=========+=========+
Ec-14_004320     -------SDSDSGDINGSDTETSSRSFSHRKSMSSSNVSSMGRRASHTFREQVRRVSQHL
MSTRG.49732.1    GGSSVGGAEEANSDDNDDDDETSCSSFSRRESRSFSQASTVGRRASVGIRRQARRVSRRV
                              ############################################   


                        790       800       810       820       830       840
                 =========+=========+=========+=========+=========+=========+
Ec-14_004320     ------ANTTKLAMDVTKATDRIVRKPSIKAKLDLFNDLSDIYPFGSPTLFHKTLDVLLL
MSTRG.49732.1    SQTAVWVHTTKLATDVTKVAGRFIRVVPEAPKLELVNDLSDIYPLSNPAIFHKTLDALLL
                         ####################################################


                        850       860       870       880       890       900
                 =========+=========+=========+=========+=========+=========+
Ec-14_004320     LSCTYLV-----------------------------------------------------
MSTRG.49732.1    LSCMYLALWAVNFMSIANEKKLAFLRHIAMLLPAAGAFLFNALSVKTSSLLLAITAMDPE
                 ######                                                      


                        910       920       930       940       950
                 =========+=========+=========+=========+=========+==
Ec-14_004320     --------RLFVSGIL-----------------------------------L
MSTRG.49732.1    IIGKVRHRELFINGPTGRMHSEDNRTRLDSPIVRAHHETRKSQVSPNTKTDY
```

```
Parameters used
Minimum Number Of Sequences For A Conserved Position: 2
Minimum Number Of Sequences For A Flanking Position: 2
Maximum Number Of Contiguous Nonconserved Positions: 8
Minimum Length Of A Block: 10
Allowed Gap Positions: None
Use Similarity Matrices: Yes
```

```
Flank positions of the 11 selected block(s)
Flanks: [1  231]  [236  247]  [264  379]  [388  443]  [447  463]  [542  564]  [572  595]  [617  629]  [685  703]  [734  777]  [789  846]  

New number of positions in Ec-14_004320.aa.fasta.trans.aa_ali.fasta-gb:  613  (64% of the original 952 positions)
```
